# *LjNRT2.3* plays a hierarchical role in the control of high affinity transport system for root nitrate acquisition in *Lotus japonicus*

**DOI:** 10.3389/fpls.2022.1042513

**Published:** 2022-11-10

**Authors:** Alessandra Rogato, Vladimir Totev Valkov, Maurizio Chiurazzi

**Affiliations:** Institute of Biosciences and Bioresources, National Research Council (CNR), Napoli, Italy

**Keywords:** nitrate, transporters, NRT2, root architecture, response to nitrogen starvation

## Abstract

Nitrate is a key mineral nutrient required for plant growth and development. Plants have evolved sophisticated mechanisms to respond to changes of nutritional availability in the surrounding environment and the optimization of root nitrate acquisition under nitrogen starvation is crucial to cope with unfavoured condition of growth. In this study we present a general description of the regulatory transcriptional and spatial profile of expression of the *Lotus japonicus* nitrate transporter *NRT2* family. Furthermore, we report a phenotypic characterization of two independent *Ljnrt2.3* knock out mutants indicating the involvement of the *LjNRT2.3* gene in the root nitrate acquisition and lateral root elongation pathways occurring in response to N starvation conditions. We also report an epistatic relationship between *LjNRT2.3* and *LjNRT2.1* suggesting a combined mode of action of these two genes in order to optimize the *Lotus* response to a prolonged N starvation.

## Introduction

Plants are sessile organisms growing under fluctuating environmental conditions with nutrient availability that may suddenly change over space and time (Oldroyd and Leyser, 2020). Nitrogen (N) in the form of nitrate is often a limiting resource for supporting plant growth and a regulated spatio-temporal pattern of expression of genes encoding nitrate transporters through the whole plant body represents a key resource to cope with local changes of nitrate conditions, providing nitrate uptake from the soil, translocation and storage/remobilization activities. Two protein families, the low-affinity Nitrate Transporter Peptide (NPF) and the high-affinity Nitrate Transporter (NRT2) play a primary role in this nitrate-related network (Wang et al., 2018). NRT2s represent small families of proton-coupled transporters including 7 and 4 members in *Arabidopsis thaliana* and *Oryza sativa*, respectively (Glass et al., 2001; Cai et al., 2008). All the NRT2 proteins characterized so far in higher plants, transport only nitrate as substrate, displaying a high affinity activity (HATS), except the *O. sativa* NRT2.4 that was reported to act as a dual affinity nitrate transporter in *Xenopus laevis* oocytes (Wei et al., 2018). The plant NRT2s transport activities depends by an additional component, NAR2/NRT3 that physically interact with NRT2s and is required for plasma membrane targeting and NRT2 stability (Kotur et al., 2012). The exceptions are represented by AtNRT2.7 and OsNRT2.4 that achieve nitrate uptake in *Xenopus* oocytes alone, without NAR2 co-expression (Chopin et al., 2007; Wei et al., 2018). The HATS for nitrate work at concentrations below 0.5 mM and it is constituted of both constitutive (cHATS) and nitrate-inducible (iHATS) components (Glass and Siddiqi, 1995; Crawford and Glass, 1998; Okamoto et al., 2003; Wang et al., 2012). The nitrate uptake activity has been reported in *Arabidopsis* for AtNRT2.1, AtNRT2.2, AtNRT2.4 and AtNRT2.5 (Li et al., 2007; Kiba et al., 2012; Lezhneva et al., 2014). AtNRT2.1 is strongly and quickly induced after addition of nitrate, playing a primary role in iHATS nitrate uptake (about 70%), whereas AtNRT2.2 makes a smaller contribution on this pathway (Filleur et al., 2001; Li et al., 2007). On the other hand, *AtNRT2.4* and *AtNRT2.5* transporters are active in the nutrient uptake without prior exposure to nitrate (cHATS) as they are induced by N-starvation conditions (Kiba et al., 2012; Lezhneva et al., 2014; Kotur and Glass, 2015). However, both the iHATS- and cHATS-mediated nitrate uptake are also involved in the primary nitrate (PNR) and the nitrogen starvation (NSR) responses that are triggered by nitrate treatment of N-depleted plants and by a prolonged N starvation condition, respectively (Hu et al., 2009; Krapp et al., 2011; Safi et al., 2021). PNR and NSR achieve a wide profile of gene expression, which includes among many, nitrate and nitrite reductases, glucose-6-phosphate dehydrogenase (G6PDH), glutamate dehydrogenase (GDH3), transcription factors (TF), other than nitrate transporters (Wang et al., 2004; Krouk et al., 2010a; Kiba et al., 2012; Marchi et al., 2013; Zamboni et al., 2014). cHATS and iHATS are likely related each other in the control of NSR and PNR pathways as the cHATS system of nitrate uptake, activated without prior exposure, is required for the immediate acquisition of the nutrient in low nitrate conditions with the consequent induction of the iHATS-mediated nitrate uptake (Behl et al., 1988).

It is well known that nitrate serves not only as a N nutrient but also as a signal molecule that regulates different plant developmental processes including, root architecture, shoot development, seed germination and flowering (Zhang and Forde, 1998; Remans et al., 2006; Krouk et al., 2010b; Nacry et al., 2013; Lin and Tsay, 2017; Safi et al., 2017). The nitrate-mediated root growth, which involves lateral root (LR) initiation, lateral root elongation, root hair growth, and primary root growth is based on local and systemic nitrate signaling pathways that are integrated through long-distance communication to orchestrate root growth in response to the uneven nitrate concentration in soil (Forde, 2014). Several players involved in the nitrate signaling pathway have been identified, including nitrate transceptors, calcium signaling, kinases and transcription factors (Wang et al., 2018). In *Arabidopsis* a crucial role in the perception of external nitrate is played by the dual-affinity transceptor *AtNPF6.3* (Remans et al., 2006; Ho et al., 2009) and *AtNRT2.1* has been also reported to act as a nitrate sensor for root development (Little et al., 2005).

The study of nitrate uptake in crops represents a hotspot in agricultural studies because of its crucial role in determining the Nitrogen Use Efficiency (NUE). Improving NUE represents a very significant challenge in agriculture in order to reduce the input costs of farming for the production of N fertilizers and to alleviate the impact of eutrophication with consequent negative effects on soil and atmospheric pollution. N uptake efficiency (NUpE) is a crucial parameter that together with N utilization efficiency (NUtE) contributes to determine the NUE in crops (Xu et al., 2012). Several studies have been focused on the attempts to improve nitrate uptake through the manipulation of the nitrate transporter genes. Direct and un-direct over-expression of both *NPF* and *NRT2* genes involved in the nitrate uptake pathways were reported to improve NUE in crops. In the case of the *NRT2* genes, over-expression of the *Oryza sativa OsNRT2.1, OsNRT2.3a* and *OsNRT2.3b* leads to increased nitrate uptake enhancing the NUE (Chen et al., 2016; Chen et al., 2017; Luo et al., 2020). Interest on nitrate transporters in legumes has recently increased because of some reports pointed to identify the role of *NPF* and *NRT2* genes in the nitrate dependent signaling pathways governing the initiation, development and functioning of the N_2_-fixing nodule (Omrane and Chiurazzi, 2009; Valkov and Chiurazzi, 2014), which represents the result of the symbiotic interaction with rhizobia (Yendrek et al., 2010; Valkov et al., 2017; Valkov et al., 2020; Wang et al., 2020; Misawa et al., 2022). Nevertheless, studies on root nitrate uptake network in legumes, the identification of the transporters involved and the investigation of their involvement with the genetic programs governing root development is lagging behind.

Here we report a molecular characterization of the *Lotus japonicus NRT2* family by analyzing the transcriptional regulatory profiles in response to N starvation conditions and by describing the spatial profile of expression of the *NRT2* and *NAR2* genes in *Lotus* roots. Furthermore, the phenotypic analysis carried out on two independents knocks out mutants indicates the involvement of *LjNRT2.3* in the root nitrate acquisition and LR elongation pathways. The possible role and mode of action of *LjNRT2.3* is discussed.

## Material and methods

### Plant material and growth conditions

All experiments were carried out with *Lotus japonicus* ecotype B-129 F12 GIFU (Handberg and Stougaard, 1992; Jiang and Gresshoff, 1997). Plants were cultivated in a controlled growth chamber with a light intensity of 200 μmol.m^-2^.sec^–1^ at 23°C with a 16 h: 8 h, light: night cycle. Seeds sterilization was performed as described in Barbulova et al. (2005). Five days after sowing on H_2_O agar Petri dishes axenic conditions, unsynchronized seedlings were discarded. Seedlings were grown on solid growth media with the same composition than Gamborg B5 medium (Gamborg, 1970) except that (NH_4_)_2_SO_4_ and KNO_3_ were omitted and substituted by the proper KNO_3_ concentrations. KCl was added, when necessary to the medium to replace the same concentrations of potassium source. The media containing vitamins (Duchefa catalogue G0415) were buffered with 2.5 mM 2-(N-morpholino) ethanesulfonic acid (MES; Duchefa, M1503.0250) and pH adjusted to 5.7 with KOH. Plant length parameters were measured with the ImageJ software (Schneider et al., 2012).

*M. loti* inoculation was performed as described in Rogato et al. (2016). The strain R7A used for the inoculation experiments is grown in liquid TYR-medium supplemented with rifampicin (20 mg/l).

### Determination of nitrate content

Tissues were first weighed and then frozen at -80°C. The frozen samples were grinded with a tissue lyser (Qiagen, 85220) at 29 Hz/for 1 min 30 sec. The powder was immediately resuspended in H_2_O (6 ml H_2_O/g of fresh weight), vortexed and centrifuged at 16.2 g to recover the supernatant. The colorimetric determination of nitrate content in leaves and roots extracts was described by Pajuelo et al. (2002). Briefly, 200 μl of 5% (w/v) salicylic acid in concentrated sulfuric acid is added to aliquots of 50 μL of crude extracts and left for 20 min at room temperature. NaOH (4.75 ml of 2N) is added to the reaction mixtures and absorbance scored at 405 nm after cooling. A calibration curve of known amount of NaNO_3_ (Sigma, 74246), dissolved in the extraction buffer is used as standard. Controls are prepared without salicylic acid.

### *L. japonicus* transformation procedures

Binary vectors were conjugated into the *Agrobacterium rhizogenes* 15834 strain (Stougaard et al., 1987). *A. rhizogenes*-mediated *L. japonicus* transformations have been performed as described in D’apuzzo et al., 2015. Inoculation of composite plants was described in Santi et al. (2003).

### Constructs preparation

Promoter-*gus*A fusions. The PCR amplified fragments containing 1000 bp (*LjNRT2.3*), 1961 bp (*LjNRT2.1*) and 1229 bp (*LjNAR2*) upstream of the ATG were obtained on genomic DNA with forward and reverse oligonucleotides containing *Sal*I (or *Hind*III) and *Bam*HI sites, respectively (Supporting Information **Table S1**). The three amplicons were sub-cloned as *Sal*I-*Bam*HI (or *Hind*III-*Bam*HI) fragments into the pBI101.1 vector to obtain translational fusions with the *gus*A marker (Jefferson, 1987).

### Quantitative Real-time RT-PCR

Real time PCR was performed with a DNA Engine Opticon 2 System, MJ Research (MA, USA) using SYBR to monitor dsDNA synthesis. The procedure is described in Moscatiello et al. (2018). The ubiquitin (*UBI*) gene (AW719589) has used as an internal standard. The oligonucleotides used for the qRT-PCR are listed in the Supporting Information **Table S1**.

### LORE1 lines analyses

LORE1 lines 30016697 and 30078926 were obtained from the *LORE1* collection (Fukai et al., 2012; Urbanski et al., 2012; Malolepszy et al., 2016). Plants in the segregating populations have been genotyped and expression of homozygous plants tested with oligonucleotides listed in the Supporting Information **Table S1**. After PCR genotyping, the clonal propagation of shoot cuts of homozygous plants in axenic conditions was performed as described in Sol et al. (2019).

### Histochemical GUS analyses

Histochemical GUS staining, fixation and sections of the root material were performed as described in Rogato et al. (2008).

### Statistical analyses

Statistical analyses were performed using the VassarStats two-way factorial ANOVA for independent samples program.

## Results

### *LjNRT2.3* is induced in response to N starvation and it is not regulated during symbiotic interaction

**Figure 1:**
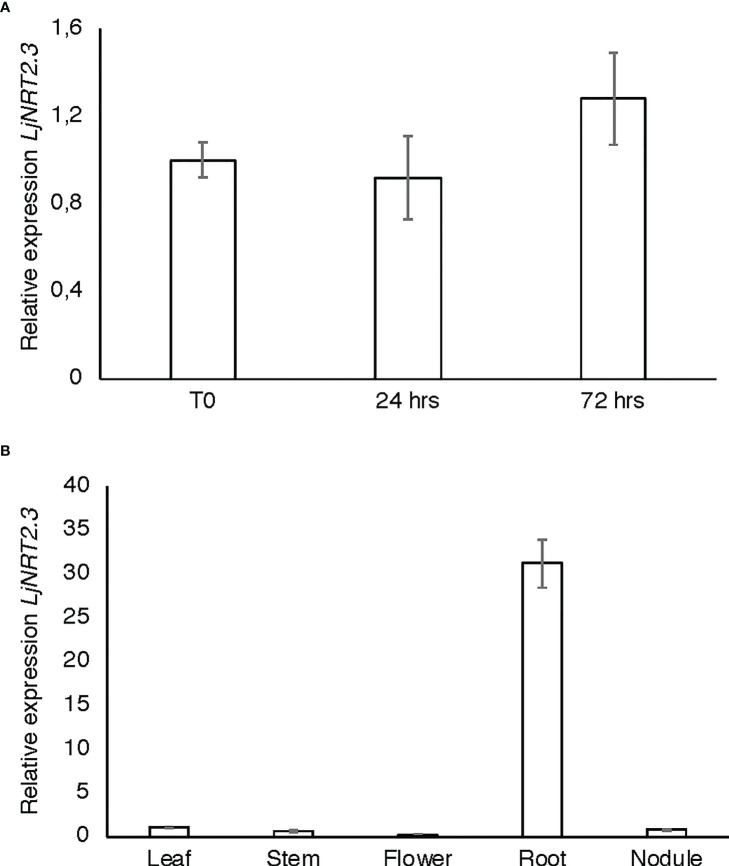
*LjNRT2.3* transcriptional regulation. **(A)** Time course analysis in wild type *L. japonicus* root and nodule tissues after *M. loti* inoculation. RNAs were extracted from roots of seedlings grown in N starvation conditions at different times after inoculation (T0, 24 hrs, 72 hrs). **(B)** Expression in different organs. RNAs were extracted at four weeks after inoculation. Mature flowers were obtained from Lotus plants propagated in the growth chamber. Expression levels obtained by qRT-PCR were normalized with respect to the internal control ubiquitin (*UBI*) gene and plotted as relative to the expression of T0 **(A)** and flowers **(B)**. Data bars represent the mean and standard deviations of data obtained with RNA extracted from three different sets of plants and 3 real-time PCR experiments.

**Figure 2:**
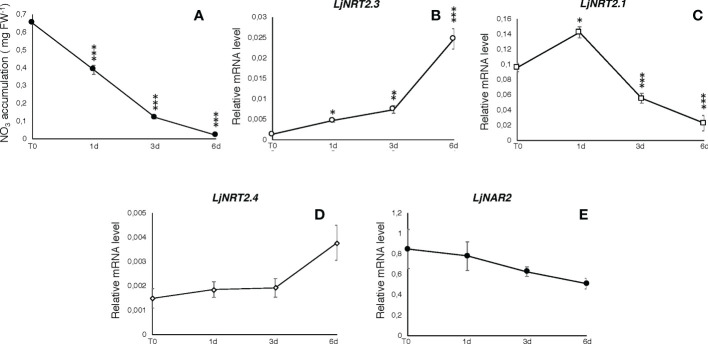
Responses to N starvation conditions. Plants were grown for 8 days on B5 derived medium supplemented with 8 mM KNO_3_ as sole N source and transferred on N starvation conditions (T0). **(A)** Measures of nitrate content in *Lotus* plants during N starvation. Samples were collected at days 0, 1, 3 and 6. Measures are means and SE of three replicates (pooling 4 plants for every time point). Asterisks indicate significant differences as relative to nitrate content at T0 (*** p<0.0001). **(B–E)** Profiles of expression of *LjNRT2* and *LjNAR2* genes in response to N starvation. RNA was extracted from roots at T0, 1, 3 and 6 days after the transfer. Expression levels obtained by qRT-PCR were normalized with respect to the internal control ubiquitin (*UBI*) gene. Data represent the mean and standard deviations of results obtained with RNA extracted from three different sets of plants and 3 real-time PCR experiments. Asterisks indicate significant differences as relative to expression at T0 (*p<0.01; **p<0.001; ***p<0.0001).

The *L. japonicus NRT2* family has been first described as constituted by four members (*LjNRT2.1-LjNRT2.4*; Criscuolo et al., 2012). More recently, the analyses provided by Misawa et al. (2022) revealed an un-accurate gene prediction for the *LjNRT2.2* gene in the *L. japonicus* MG20 ecotype (Lj3g3v3069050.1; http://www.kazusa.or.jp/lotus/index.html), with a premature stop codon determining a truncated version of the protein. This result was confirmed in the *L. japonicus* GIFU ecotype indicating an independent evolution of this gene in these *Lotus* ecotypes (Misawa et al., 2022). Therefore, the *NRT2* family of *L. japonicus* is constituted by three functional genes. The name *LjNRT2.3* has been assigned to the MG20 gene *Lj4g3v1085060.1* and to the identical copy *LotjaGi4g1v0155900.1*, identified in the *L. japonicus* accession Gifu (Valkov et al., 2020; https://lotus.au.dk/). *LjNRT2.3* encodes for a 507 amino acid protein with a molecular mass of 55.4 kDa and 11 TM predicted domains (**Supplementary Figure S1**; Tusnády and Simon, 2001). The two members of the *L. japonicus NRT2* family previously characterized, *LjNRT2.1* and *LjNRT2.4* have been reported to play important roles in different steps of the nodulation program. Consistently, their profiles of expressions revealed a clear-cut relationship with the nodulation programme Misawa et al., 2022) as *LjNRT2.4* shows a significant induced expression in the nodule organ (Valkov et al., 2020) and *LjNRT2.1* is significantly expressed in the root cortical region, at early times after *Mesorhizobium loti* inoculation (Misawa et al., 2022). However, the relative expression profile of *LjNRT2.3* is not regulated in the root and nodule tissues during the symbiotic interaction with *M. loti* (**Figures 1A, B**). Furthermore, the *LjNRT2.3* transcript distribution in different *L. japonicus* organs indicated a substantial root-related expression profile (**Figure 1B**). LjNRT2.3 shares the highest level of amino acid identity with the AtNRT2.5 protein (74%). *AtNRT2.5* (AT1G12940.1) was reported to be strongly induced in *A. thaliana* plants grown under nitrogen starvation (Lezhneva et al., 2014; Kotur and Glass, 2015). In the preliminary molecular characterization of *LjNRT2.3* reported in Criscuolo et al. (2012), we have shown a quick induction of expression in roots of *Lotus* plants transferred in a medium supplemented with low KNO_3_ concentrations (0.01 and 0.1 mM) as compared to plants transferred in sufficient KNO_3_ conditions (1 and 2 mM). In order to better characterize the regulatory profile of *LjNRT2.3*, *L. japonicus* plants grown for 10 days in the presence of 8 mM KNO_3_ were transferred on a growth medium without N sources and a time course of expression was conducted with RNAs extracted from root tissue. As indicated in **Figure 2A**, the nitrate content in the *Lotus* plants decreased quickly and progressively after the transfer in N starvation conditions. The amount of the *LjNRT2.3* transcript increased rapidly and strongly up to six days after transfer (23 fold; **Figure 2B**). This pattern of expression was unique for the *LjNRT2* family as *LjNRT2.1* displayed a progressive reduced level of expression, although a significant level of expression was maintained at the end of the N-starvation period (**Figure 2C**). *LjNRT2.4* was only slightly induced (2.5 fold) after 6 days of N starvation (**Figures 2C, D**). We have also included in this transcriptional analysis the gene encoding for the activating partner protein NAR2 that is required for plasma membrane targeting and NRT2 stability (Kotur et al., 2012). A blast search in the *Lotus* genome database has led to the identification of a single homologous protein sharing 55% of amino acid identity with the *Arabidopsis* AtNAR2.1 protein that was named LjNAR2 (LotjaGi4g1v0227700.1). The profile of expression of *LjNAR2* was unresponsive to N-starvation conditions (**Figure 2E**).

### *LjNRT2.3* is expressed in the root hairs and epidermal cell layer

**Figure 3:**
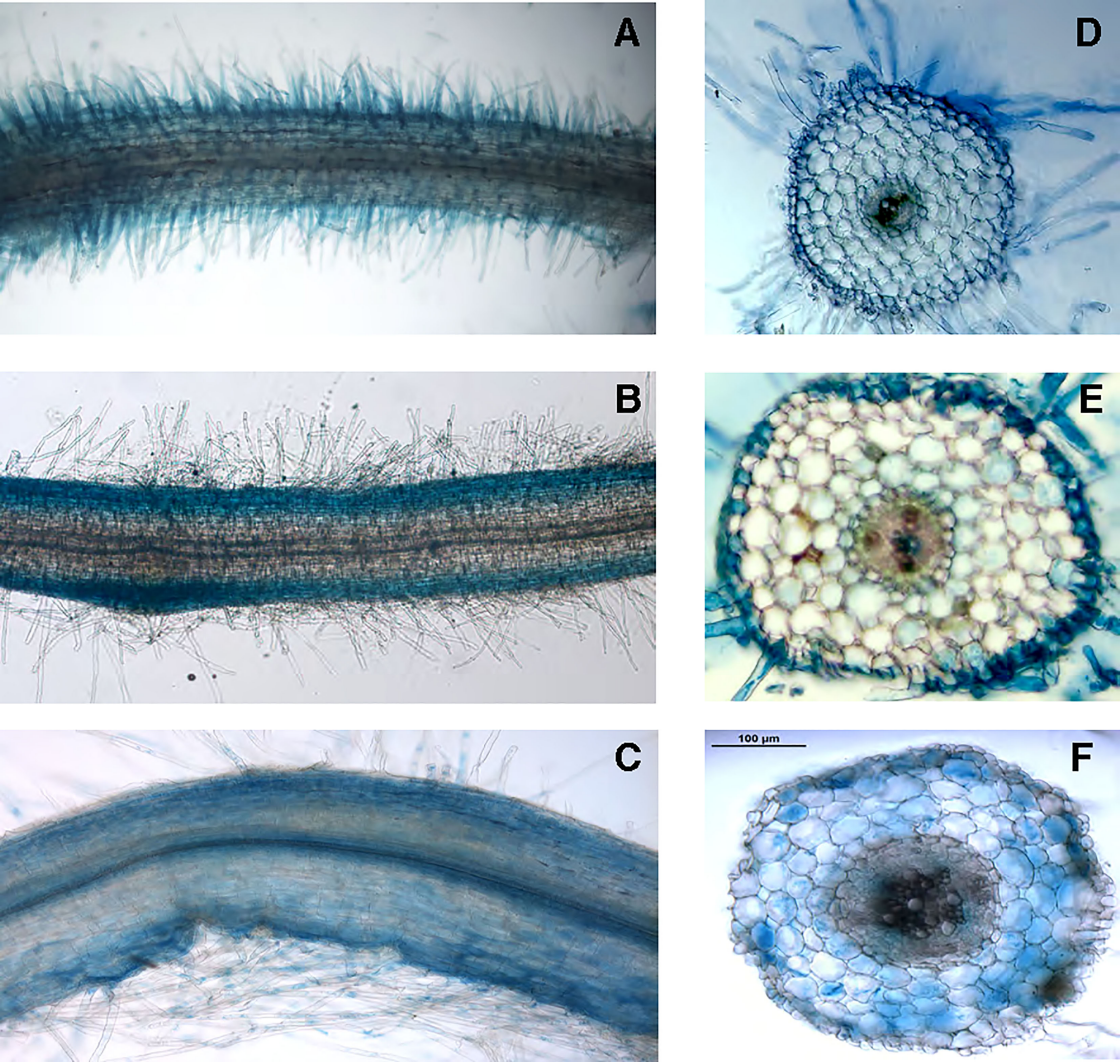
Representative GUS activity of *L. japonicus* transgenic hairy roots transformed with the pr*LjNRT2.3*- **(A, D)**, pr*LjNRT2.1*- **(B, E)** and pr*LjNAR2*-*gus*A **(C, F)** constructs. **(A–C)** whole mount root samples. **(D–F)** 70 μm root cross sections.

To gain further information about the physiological role played by *LjNRT2.3* we have analyzed and compared the patterns of spatial distribution of GUS activity obtained in transgenic hairy roots transformed with the *LjNRT2.3*, *LjNRT2.1* and *LjNAR2* promoter-*gus*A fusions. The *LjNRT2.3* expression was subtly confined to the epidermal root cell layer with a strong GUS activity detected in the root hairs (**Figures 3A, D**). This profile of spatial expression overlaps the one obtained for *LjNRT2.1* (**Figures 3B, E**), which confirmed the pattern reported by Misawa et al. (2022). Interestingly, the *LjNAR2* expression was not confined to the epidermis but also detected in the cortical root region (**Figures 3C, F**). The profile of expressions shown in **Figure 3** suggests a role of *LjNRT2.3* in enhancing nitrate uptake in the roots, although the data shown in **Figure 2B** indicate that such a function, which would be shared with *LjNRT2.1*, may be played in response to specific N starvation environmental conditions. The spatial pattern of expressions of the *NRT2* genes in *L. japonicus* is completed by the one already reported for *LjNRT2.4* that is specifically expressed in the root vascular structures and hence appears to not directly participate to the root nitrate uptake from the soil (Valkov et al., 2020).

### Isolation of *Ljnrt2.3* null mutants and phenotypic characterization

**Figure 4:**
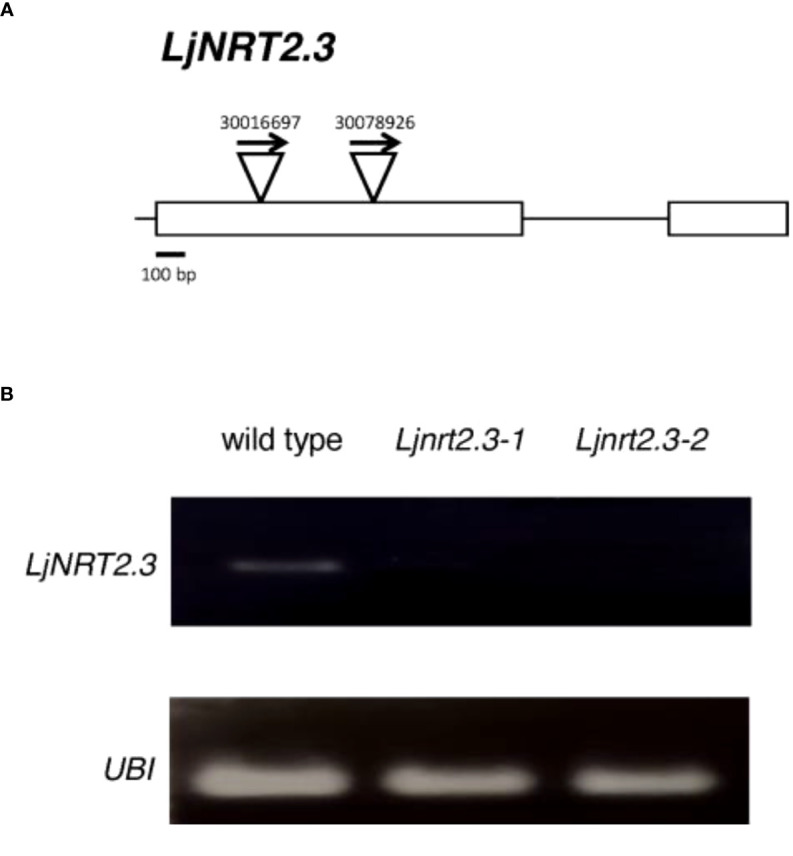
**(A)** Exon/intron organization of the *LjNRT2.3* gene. Insertion sites, relative orientations of the LORE1 retrotransposon element in the 30016697 and 30078926 lines are indicated. **(B)** *LjNRT2.3* is not expressed in the LORE1 homozygous mutant lines *Ljnrt2.3-1* and *Ljnrt2.3-2*.

**Figure 5:**
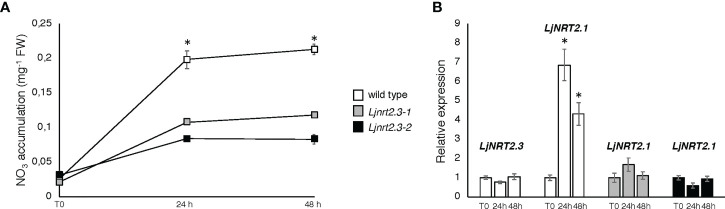
Plants were grown for 8 days on B5 derived medium supplemented with 8 mM KNO_3_ as sole N source and then transferred under N starvation conditions for 6 days. At the end of the *LjNRT2.3* induction period, plants were transferred on the same medium supplemented with 0.5 mM KNO_3_ (T0) to score nitrate content and expression profiles at 24 and 48 hrs. **(A)** Nitrate content in roots of wild type and *Ljnrt2.3* plants. Data represent means and SE from three independent experiments (8 plants per experiment). Asterisks indicate significant differences (p<0.001) of nitrate content in wild type and *Ljnrt2.3-1* plants. **(B)** Profiles of *LjNRT2.1* and *LjNRT2.3* expression in roots of wild type and *Ljnrt2.3* plants in response to 0.5 mM KNO_3_ repletion. Data bars represent means and SD from three independent experiments. Asterisks indicate significant differences as relative to expression at T0 (*p<0.0001). The plant genotypes are indicated.

In order to investigate the physiological function of *LjNRT2.3*, two independent LORE1 insertion mutants have been isolated from the *L. japonicus* LORE1 lines collection (Fukai et al., 2012; Urbanski et al., 2012; Malolepszy et al., 2016). Lines 30016697 and 30078926, bearing retrotransposon insertions in the first exon (**Figure 4A**), were genotyped by PCR. Endpoint RT-PCR analyses on RNAs extracted from roots of homozygous plants from lines 30016697 and 30078926 (hereafter called *Ljnrt2.3-1* and *Ljnrt2.3-2*, respectively) revealed no detectable *LjNRT2.3* full size mRNA and hence, considered null mutants (**Figure 4B**). In order to test whether the induced pattern of expression observed for *LjNRT2.3* under N starvation conditions (**Figure 2B**) represents a pre-requisite for an efficient nitrate uptake in *Lotus* roots after the transfer in the presence of low nitrate concentrations, we have compared the nitrate accumulation in roots of wild type and mutant plants. Plants have been grown as for the experimental scheme described in **Figure 2**. After 6 days of growth in a medium without N sources, wild type and *Ljnrt2.3* plants were transferred in the same medium supplemented with 0.5 mM KNO_3_ and the root nitrate content was measured in a time course experiment. A clear-cut reduction in NO_3_^-^ content was scored in both the *Ljnrt2.3-1* and *Ljnrt2.3-2* roots as compared to wild type at 24 and 48 hrs after the transfer, strongly suggesting the involvement of *LjNRT2.3* in root nitrate acquisition (**Figure 5A**). This was also confirmed by the profile of expression of *LjNRT2.3* that was maintained constant up to 48 hrs after the transfer in the presence of 0.5 mM KNO_3_ (**Figure 5B**). Moreover, we have also evaluated the involvement of *LjNRT2.1*, which shares with *LjNRT2.3* the spatial pattern of expression in the epidermal cell layer (**Figures 2A–D**). *LjNRT2.1* has been recently reported to be involved in the root nitrate uptake at both low (0.2 mM) and high (10 mM) KNO_3_ conditions and this was also correlated to a reduced nitrate content in the roots of mutants grown in the presence of 10 mM KNO_3_ as compared to wild type (Misawa et al., 2022). Interestingly, the qRT-PCR results shown in **Figure 5B** indicates a significant induction of *LjNRT2.1* in wild type roots after the transfer in the presence of 0.5 mM KNO_3_ (up to 7-fold at 24 hrs; **Figure 5B**) and this profile was completely abolished in the *Ljnrt2.3* genetic background (**Figure 5B**).

### *LjNRT2.3* is required for LR elongation under low nitrate conditions

**Figure 6:**
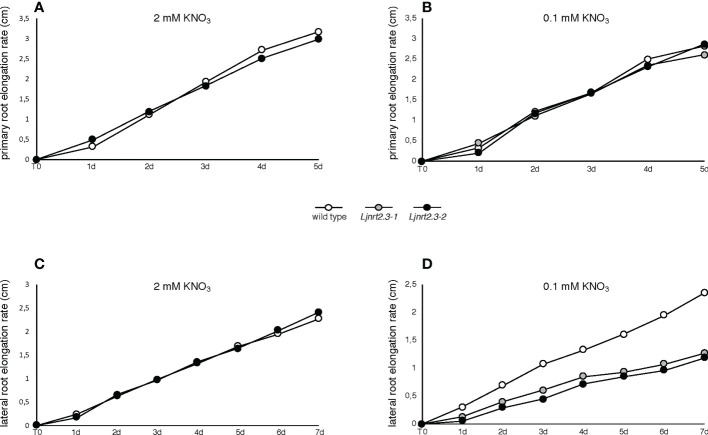
Root elongation rates in wild type and *Ljnrt2.3* plants. **(A, B)** elongation rate of primary roots of wild type and *Ljnrt2.3-1* plants. **(C, D)** elongation rate of lateral roots of wild type, *Ljnrt2.3-1* and *Ljnrt2.3-2* plants. Synchronized seedlings were grown for 8 days on B5 derived medium supplemented with 8 mM KNO_3_ as sole N source and then transferred under N starvation conditions for 6 days. Then plants were transferred on 0.1 mM and 2 mM KNO_3_ (T0) and measures of primary and lateral roots taken every day. The scoring was carried out on lateral roots long at least 0,3 cm at the time of the transfer. The KNO_3_ concentrations and plant genotypes are indicated. Wild type and *Ljnrt2.3* plants were grown in the same square Petri dishes to minimize the differences of growth conditions. Data represent means of measures performed in two/three independent experiments (18 plants per experiment per condition). A significant difference (p<0.001) was obtained for lateral roots of the *Ljnrt2.3* plants scored from T0 to 7d in 0.1 mM KNO_3_.

In addition to its role as nutrient, nitrate has been proved to act as a signal regulating many physiological processes, including root architecture. The results reported in **Figure 5A** prompted us to investigate whether the changes scored in the nitrate acquisition capacity of the *Ljnrt2.3* mutants could also determine an alteration of the signaling pathways controlling the root system developmental programs. Therefore, the experimental scheme adopted to maximize the expression of the *LjNRT2.3* gene has been exploited to check whether the root elongation programme was affected in the *Ljnrt2.3* knock out background. After the induction treatment (6 days of N starvation conditions) plants have been transferred under two different KNO_3_ conditions, 0.1 mM and 2 mM and the kinetics of elongation of primary root and LRs scored for 5 and 7 days, respectively. Wild type and mutant plants did not exhibit evident shoot growth phenotypes at the end of this cycle of growth (**Supplementary Figure S2**). Nevertheless, this experimental system allowed to reveal striking differences in the kinetics of root elongation. The kinetics of elongation of the primary roots in both nitrate conditions were quite similar and constant (about 0.5 cm/day) and no differences were revealed between wild type and *Ljnrt2.3* plants (**Figures 6A, B**). In the same way, the mean of the kinetics of LR elongation remained constant in wild type and mutant plants in the presence of 2 mM KNO_3_ (**Figure 6C**; about 0.3 cm/day). Interestingly, the kinetics of elongation of LR scored in 0.1 mM KNO_3_ conditions, showed a significant difference in both *Ljnrt2.3-1* and *Ljnrt2.3-2* plants as compared to wild type with a progressive slowdown of the elongation rate (**Figure 6D**; 0.32 cm/day vs 0.18 cm/day).

## Discussions

The distribution of *NRT2* families in land plants is very patchy with a number of members that does not linearly fits with the level of ploidy and overall genome size in different monocot and dicot plants. The number of *NRT2* genes spans from 33 in *Triticum aestivum*, 17 in *Brassica napus*, 7 in *A. thaliana*, 5 in *O. sativa*, 6 in *Populus trichocarpa*, 6 in *Manihot esculenta*, 4 in *Hordeum vulgare*, 4 in *Solanum lycopersicon*, 2 in *Cucumis sativus*, 9 in *Physcomitrella patens* (Zoghbi-Rodrìguez et al., 2021; Akbubak et al., 2022). The situation is even more intriguing in legumes where the reported number of *NRT2* genes is generally low: 3 in *L. japonicus* (Misawa et al., 2022), 3 in *M. truncatula* (Pellizzaro et al., 2015), 5 in *Glycine soya* (You et al., 2020), 1 in *Pisum sativum* (Gu et al., 2022). It was recently hypothesized that the decreased number of *NRT2* genes and less reliance on NO_3_^-^ in legumes could be related to the evolution of the supplying capacity from N-fixing nodules (Gu et al., 2022). This heterogenicity makes difficult the identification of possible functional orthologues among the NRT2 proteins of various plants with the exception of AtNRT2.5, where phylogenetic analyses have revealed the existence of closely related orthologues in both dicots and monocots with the exception of Solanaceae (Plett et al., 2010; Kotur and Glass, 2015). However, functional divergency may have evolved for NRT2.5s in different species and environments as for alophyte and non-alophyte plants (Liu et al., 2020). We have already reported the phylogenetic analysis that places the *LjNRT2.3* gene in the *NRT2.5* sub-group (Valkov et al., 2020). The profile of expression displayed by *LjNRT2.3* in different organs (**Figure 1B**) resembles the one reported for *AtNRT2.5* and *OsNRT2.3a* with a preferential expression in roots (Tang et al., 2012; Lezhneva et al., 2014). However, the preferential expression in roots is not a constant feature in different plant species as in some monocots the *NRT2.5* transcript level is increased in the shoot (Lezhneva et al., 2014). *NRT2.5* expression has been also reported in embryos and shell in wheat (Sabermanesh et al., 2017) as well as cobs in corn indicating a role in the accumulation of nitrate in seed and filling of the grain (Ju et al., 2019). However, *LjNRT2.3* expression in seed tissue is not reported in the RNAseq data of the *Lotus* expression atlas (https://lotus.au.dk/expat/). On the contrary, what seems to represent an evolutionary conserved mark in most plant species is the increase in the *NRT2.5*-like gene expressions in roots in response to N starvation (Lezhneva et al., 2014; Guo et al., 2014). This regulatory profile has been also confirmed in our experimental conditions for the *LjNRT2.3* gene (**Figure 2B**). Interestingly, the *LjNRT2.3* and *LjNRT2.1* genes displayed a complementary response to N starvation in roots with the latter showing a significant progressive reduction (up to seven-fold) in the level of transcription after a transient slight increase (**Figure 2C**). The same regulatory profile has been reported for *AtNRT2.1* in adult plant (Lezhneva et al., 2014). The profiles of expressions of *LjNRT2.4* and *LjNAR2* genes did not change significantly in response to N starvation in *Lotus* roots with the latter still maintaining the highest level of transcription (**Figures 2B–E**). The analysis in transgenic hairy roots transformed with the pr*LjNRT2.3*-*gus*A fusion indicated a spatial profile of expression for *LjNRT2.3* confined to epidermis and root hairs of primary and lateral roots (**Figure 3A**), suggesting a nitrate root uptake function. Consistently, this spatial profile of expression has been also reported for *AtNRT2.5*, which plays a role on high affinity nitrate acquisition in roots (Lezhneva et al., 2014; Kotur and Glass, 2015). Moreover, *AtNRT2.5* is also expressed in the phloematic root tissue playing a role on nitrate loading and mobilization to the shoot under conditions of N starvation (Lezhneva et al., 2014). A similar function was reported for *OsNRT2.3a* that is mainly expressed in the xylem parenchyma cells of the root stele being involved in the transport of nitrate from root to shoot under low NO_3_^-^ conditions (Tang et al., 2012). Interestingly, *LjNAR2* is spatially co-expressed with *LjNRT2.3* in the epidermal cell layer (**Figure 3**), as expected for a partner protein that facilitates the plasma membrane localization of NRT2s. The description of the pattern of spatial expression of the *Lotus NRT2* genes in **Figure 3** is completed with that of *LjNRT2.1*, which is also confined to the epidermal cell layer in agreement with the recently reported involvement on nitrate uptake in both low and high nitrate conditions (Misawa et al., 2022). To our knowledge, this is the first report of the spatial pattern of expression of the whole *NRT2* family members in legumes. In *M. truncatula* the *MtNRT2.3* gene, orthologue of *LjNRT2.3* (83% amino acid identity; Valkov et al., 2020), shows an induced expression in nodules (Pellizzaro et al., 2015). Interestingly, this nodule-induced expression profile is not observed for *LjNRT2.3* (**Figure 1**) but it is shared in *L. japonicus* by the *LjNRT2.4* gene (Criscuolo et al., 2012; Valkov et al., 2020).

**Figure 7:**
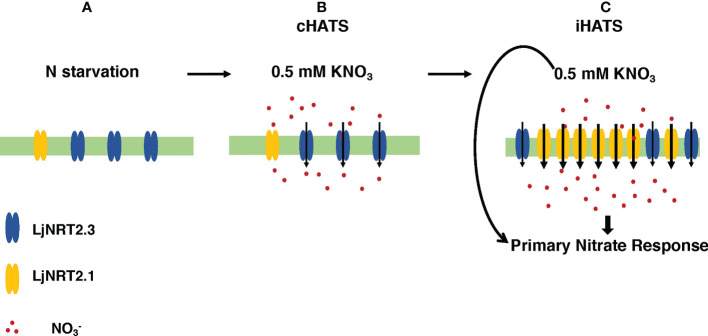
Hypothetical model of the combined action exerted by *LjNRT2.3* and *LjNRT2.1* to achieve the iHATS and the primary nitrate response after prolonged N starvation conditions. Plasma membrane localization and uptake activity of LjNRT2.3 are based on data reported for the orthologous AtNRT2.5 (Kotur et al., 2012; Lezhneva et al., 2014). **(A)** Induction of *LjNRT2.3* under N starvation conditions. **(B)** *LjNRT2.3*-mediated cHATS. **(C)** *LjNRT2.1*-mediated iHATS and triggering of the primary nitrate response. Arrows in **(C)** indicate local and systemic nitrate-mediated signalling pathways.

The phenotypic characterization carried out with two independent *Ljnrt2.3* knock out mutants indicated a significant reduction of nitrate content in the roots of mutants exposed to low nitrate conditions after a prolonged (six days) N starvation treatment as compared to wild type (**Figure 5A**). As expected the *LjNRT2.3* transcript level did not change in wild type plants transferred in the presence of 0.5 mM KNO_3_, whereas the *LjNRT2.1* expression was strongly induced by this treatment (**Figure 5B**). A quick nitrate dependent induction of expression for *LjNRT2.1* in *Lotus* roots was previously reported after exposure to both low and high nitrate conditions (Criscuolo et al., 2012; Misawa et al., 2022). However, the *LjNRT2.1* induced profile of expression is completely abolished in the *Ljnrt2.3* genetic background (**Figure 5B**). An epistatic relationship between these *NRT2* genes has been already suggested in *Arabidopsis* by the analysis carried out by Kotur and Glass (2015) where the *AtNRT2.1* expression was significantly reduced in the *Atnrt2.5* mutants after treatment with 1 mM KNO_3_. The presence in *L. japonicus* of the *LjNRT2.1* and *LjNRT2.3* as the only two genes involved in nitrate uptake in the high affinity range facilitates the understanding of their mutual functional relationship. In fact, it can be excluded as in *A. thaliana*, a role in the control of HATS due to a compensatory up-regulation of other genes such as *AtNRT2.2* in the *Atnrt2.1* genetic background (Li et al., 2007). The 60% reduction of nitrate content observed in the roots of the *Ljnrt2.3* mutants can be explained by a combined action of the *LjNRT2.3* and *LjNRT2.1* genes on the nitrate uptake mediated by the HATS in response to N starvation conditions. In particular, the expression of *LjNRT2.3* in roots exposed to N starvation may warrant the quick initiation of the nitrate uptake and assimilation pathway to set in motion the *LjNRT2.1*-mediated nitrate HATS pathway (Behl et al., 1988; Kotur and Glass, 2015) is schematized in the model hypothesized in the **Figure 7**. Consistently with this model, AtNRT2.1 has been reported as the major contributor to the iHATS activity with a nitrate dependent induced expression in Arabidopsis (Filleur et al., 2001; Okamoto et al., 2003; Li et al., 2007), but a significant reduction in the cHATS-mediated NO_3_^-^ influx has been also reported for *Atnrt2.5* mutants in adult plants grown for 10 days under N starvation conditions and then transferred in the presence of 0.2 mM KNO3 (Lezhneva et al., 2014). The partial nitrate acquisition scored at 24 hrs in the roots of the *Ljnrt2.3* plants (40% as compared to wild type roots) and maintained until 48 hrs (**Figure 5A**) could be achieved by *LjNRT2.1*, which still exhibits a significant, basic level of expression at the end of the period of N starvation (**Figure 2C**). The cHATS provided by *LjNRT2.3* is likely characterized by higher affinity and lower capacity for nitrate (Siddiqi et al., 1990; Kronzucker et al., 1995; Glass and Siddiqi, 1995; Crawford and Glass, 1998), hence warranting a more efficient and quick plant response in the presence of extremely low amounts of available nitrate in the rhizosphere.

The phenotypic characterization carried out with the *Ljnrt2.3-1* plants indicates a very intriguing role of *LjNRT2.3* gene on the LR elongation program when plants are transferred in the presence of low KNO_3_ concentrations (0.1 mM) after a prolonged N starvation treatment. The LR elongation rate of the *Ljnrt2.3-1* mutants is half that of wild type plants and this difference is not scored in the presence of 2 mM KNO_3_ (**Figures 6C, D**). The nitrate-related LR development pathway presents two major features in many plant species: *i)* a systemic repression of LR growth in the presence of high nitrate supply; *ii)* a local stimulation of LR growth by exogenous NO_3_^-^ supply. In *A. thaliana*, a localized patch of NO_3_^-^ in a split agar plate triggers a preferential LR growth in the region of the patch due to both an increase of LR elongation and stimulation of LR primordia emergence in the nitrate enriched region (Zhang and Forde, 1998; Zhang et al., 1999; Linkohr et al., 2002; Remans et al., 2006; Krouk et al., 2010b). When roots encounter a NO_3_^-^ patch of soil, a rapid physiological response is achieved, consisting of a localized and transient increase in NO_3_^-^ uptake associated to a slower developmental response characterized by higher rates of LR proliferation within the patch (Forde, 2002). Local and systemic nitrate signaling needs to be integrated through long-distance communication to control root growth in response to the variegated nitrate concentration in soil. Therefore, LR development is controlled by a very intricated network involving NO_3_^-^ uptake and signaling. In particular, NO_3_^-^ induces transcription of its own transport and assimilation pathways and of several players involved in the nitrate signaling pathway, including nitrate transceptors, regulators of calcium signaling, kinases, transcription factors, and various peptides (Omrane et al., 2009; Krouk et al., 2010a; Bellegarde et al., 2017). Finally, strong bodies of evidence indicate that many adaptive developmental responses to changes in N availability are triggered by a cross talk between N and hormone signaling pathways. Plants normally develop a more exploratory root system with longer LRs also under N deficiency (Lopez-Bucio et al., 2003; Ruffel et al., 2011) and the effect of N deprivation or low N on root branching depends on the plant nitrogen nutritional condition and the of plant stress (Krouk et al., 2010b; Forde, 2014).

The epistatic relationship between *LjNRT2.3* and *LjNRT2.1* and hence the reduced expression of *LjNRT2.1* observed in the *Ljnrt2.3* background could also explain the defect of LRs elongation rate displayed by the *Ljnrt2.3* mutants (**Figure 6D**). Consistently, *Atnrt2.1* mutants grown on vertical agar plates supplied with 0.25 mM KNO_3_ show a reduced LR growth and this phenotype, which is not scored in the presence of 2.5 mM KNO_3_ (Li et al., 2007), was associated to a significant reduction of the nitrate uptake (Li et al., 2007). Similar results have been obtained in cucumber and rice where knock-down and overexpressing *OsNRT2.1* plants, show a reduction and increase of the nitrate-dependent root elongation by regulating auxin transport to roots, respectively (Li et al., 2018; Naz et al., 2019). Interestingly, in common wheat the over-expression of the *Triticum aestivum TaNRT2.5* gene triggers higher nitrate accumulation and improved NUE, which was also associated to an increased root elongation capacity (Li et al., 2020). Moreover, a reduction of LR growth has been also reported for *Atnrt2.5* mutants that abolish the positive effect due to inoculation with the plant growth promoting bacterium strain *Phyllobacterium brassicacearum* (Kechid et al., 2013).

In conclusion, our research demonstrates the hierarchical role played by *LjNRT2.3* in the orchestration of the response achieved to optimize the nitrate acquisition in *Lotus* roots facing low nitrate concentrations after a prolonged period of N starvation. *LjNRT2.3* acts as a sentinel gene induced without prior exposure to nitrate that plays an epistatic control on the *LjNRT2.1* expression.

## Data availability statement

The datasets presented in this study can be found in online repositories. The names of the repository/repositories and accession number(s) can be found in the article/**Supplementary Material**.

## Author contributions

All the authors critically discussed results and development of the work. AR and VV have designed and performed the experiments. MC has designed the experiments and written the manuscript. All authors contributed to the article and approved the submitted version.

## Funding

This work was supported by CNR project FOE-2019 DBA.AD003.139 and within the Agritech, National Research Center and received funding from the European Union Next Generation EU, Piano Nazionale di Ripresa e Resilienza (PNRR) – Missione 4, Componente 2, Investimento 1.4 - D.D.1032, 17/06/2022, CN00000022. This manuscript reflects only the author’s views and opinions, neither the European Union nor the European Commission can be considered responsible for them.

## Acknowledgments

We thank Marco Petruzziello, Francesca Segreti and Giuseppina Zampi for technical assistance.

## Conflict of interest

The authors declare that the research was conducted in the absence of any commercial or financial relationships that could be construed as a potential conflict of interest.

## Publisher’s note

All claims expressed in this article are solely those of the authors and do not necessarily represent those of their affiliated organizations, or those of the publisher, the editors and the reviewers. Any product that may be evaluated in this article, or claim that may be made by its manufacturer, is not guaranteed or endorsed by the publisher.

## Supplementary material

Supplementary Figure 1
**(A)** Amino acid sequence of LjNRT2.3. **(B)** TMHMM prediction of LjNRT2.3 (Tusnády and Simon, 2001).

Supplementary Figure 2
**(A)** Representative images of wild type and *Ljnrt2.3-1* plants. Growth conditions: 10 days on 8 mM KNO_3_, 6 days on N starvation, 7 days on 0.1 mM or 2 mM KNO_3_. **(B)** Measures of shoot length per plant at the end of the period of growth (two independent experiments, 18 plants per experiment per condition). **(C)** Measures of shoot fresh weight per plant at the end of the period of growth.
