# Supplementary material for: LjNRT2.3 plays a hierarchical role in the control of high affinity transport system for root nitrate acquisition in Lotus japonicus
Source: Front Plant Sci. 2022 Nov 10;13:1042513. doi: 10.3389/fpls.2022.1042513 (PMC9687105; doi:10.3389/fpls.2022.1042513)

A

MDLELPNLSANESQQPKFTLPVDSENKATEFRLFSLAKPHMRSFHLSWFSFFACFVSSFA
AAPLVPIIRDNLNLTATDVGNAGVASVSGAVFARIVMGTACDLFGPRLASASLIFLTAPA
VYCTSIISSANSYLLVRFFTGFSLATFVSTQFWMSSMFSTNVVASANGLAGGWGNLGGGA
TQLIMPLVFSLIQDFGATKFTAWRIAFFIPAFLQMLTAYFILVFGQDMPDGNYHRLEKSG
DKPKDELSKVVYHGVTNYRAWILALTYGYCFGVELTIDNIIAEYFYDRFNLKLHTAGIIA
ASFGLANIFSRPGGGYVSDVMARRFGMRGRLWILWLTQTLAGVLCIIMGLVGSLSVSIIV
MIIFSVFVQAACGMTFGIVPFVSRRSLGVISGMTGGGGNVGAVVTQLIFFKGSRFSKERG
ITLMGAMIIICTLPICLIYFPQWGGMFLGPSSKKVTEEDYYMSEWNSKEQQKGSHHASLK
FADNSTSERGRKHNGATRPAEITPPHV

B

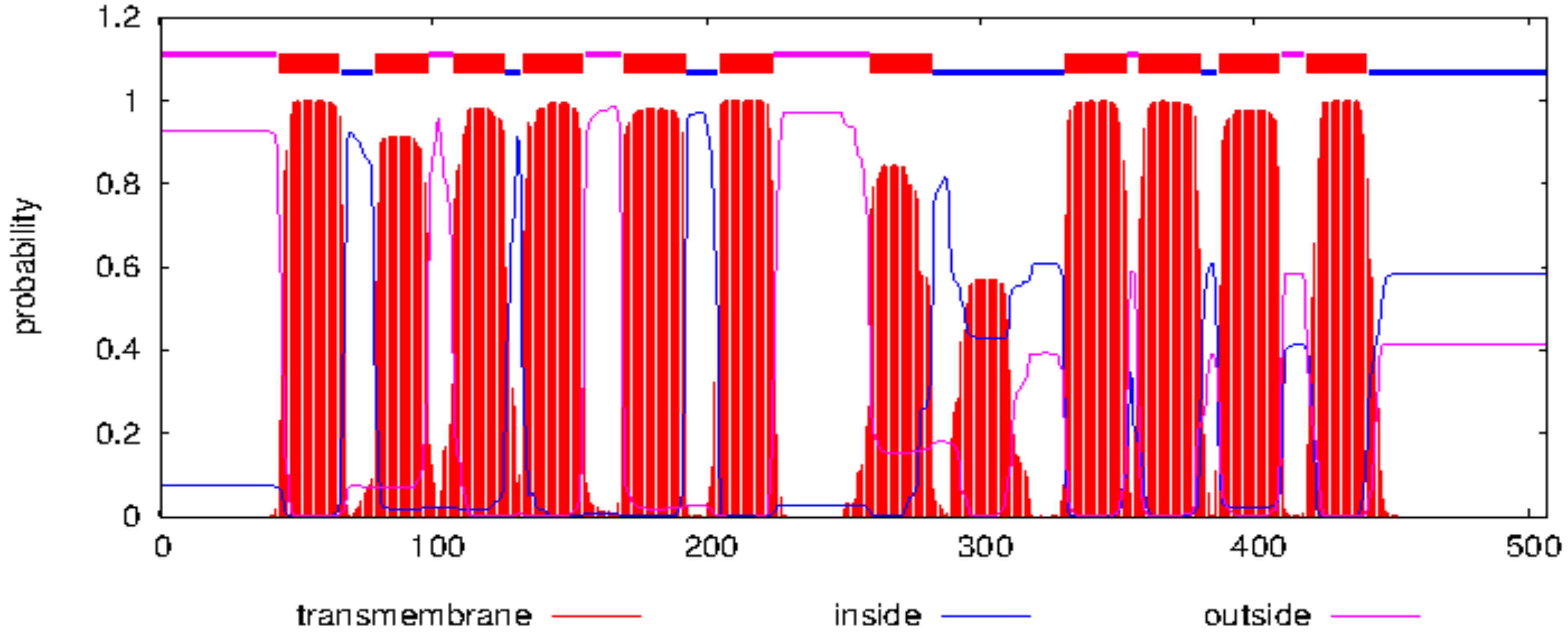

Supplement: Supplementary Figure 1 — (A) Amino acid sequence of LjNRT2.3. (B) TMHMM prediction of LjNRT2.3 (Tusnády and Simon, 2001). [file Image_1.pdf]
