# Supplementary figures and images for: LjNRT2.3 plays a hierarchical role in the control of high affinity transport system for root nitrate acquisition in Lotus japonicus
Source: Front Plant Sci. 2022 Nov 10;13:1042513. doi: 10.3389/fpls.2022.1042513 (PMC9687105; doi:10.3389/fpls.2022.1042513)

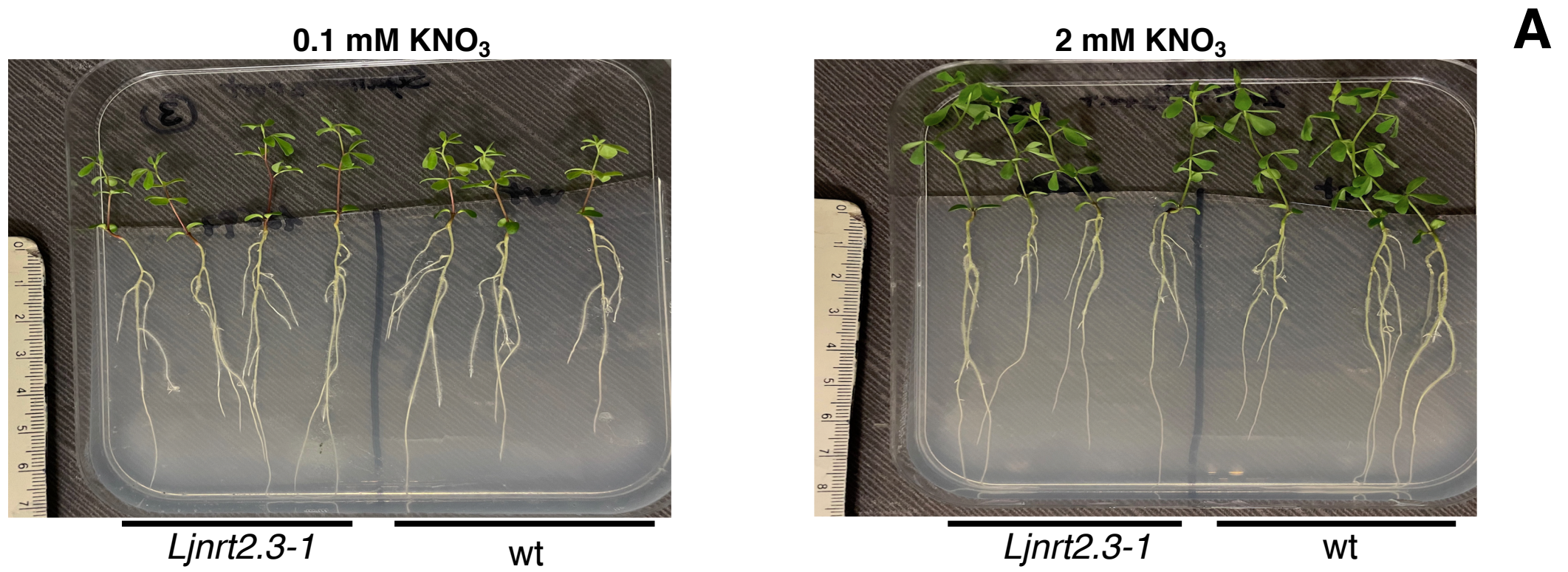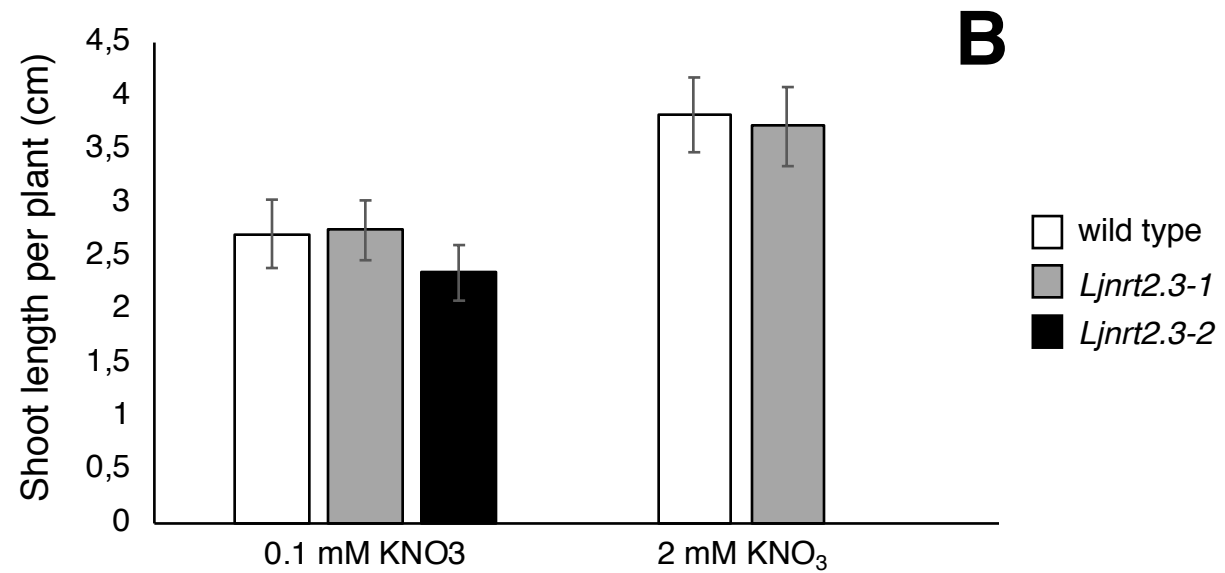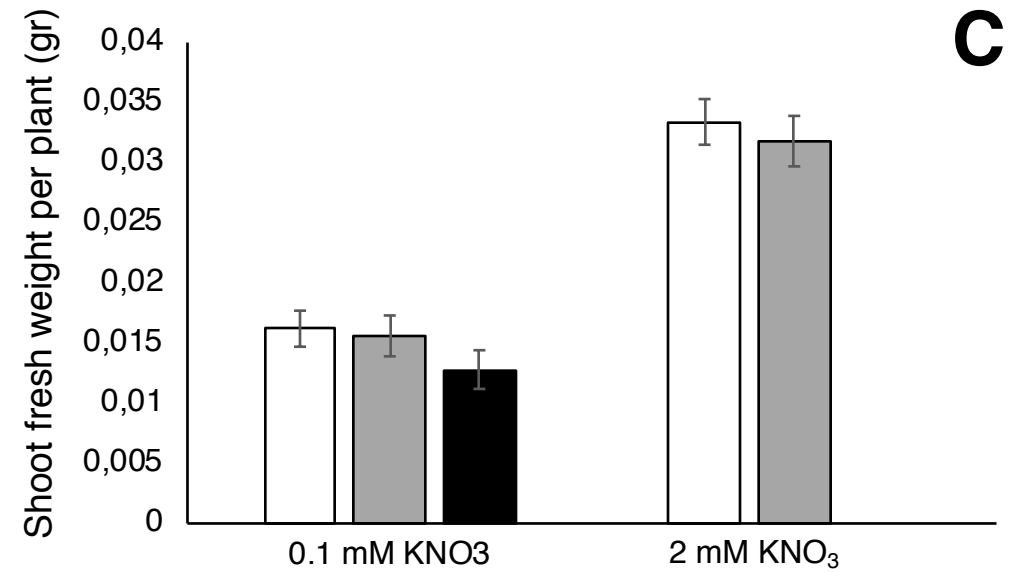

Supplement: Supplementary Figure 2 — (A) Representative images of wild type and Ljnrt2.3-1 plants. Growth conditions: 10 days on 8 mM KNO3, 6 days on N starvation, 7 days on 0.1 mM or 2 mM KNO3. (B) Measures of shoot length per plant at the end of the period of growth (two independent experiments, 18 plants per experiment per condition). (C) Measures of shoot fresh weight per plant at the end of the period of growth. [file Image_2.pdf]
